# Antioxidant Foods and Cardiometabolic Health

**DOI:** 10.3390/antiox11040746

**Published:** 2022-04-08

**Authors:** Silvia M. Arribas, María A. Martín-Cabrejas

**Affiliations:** 1 Department of Physiology, Faculty of Medicine, Universidad Autónoma de Madrid, C/Arzobispo Morcillo 2, 28029 Madrid, Spain; 2 Department of Agricultural Chemistry and Food Science, Faculty of Sciencies, Universidad Autónoma de Madrid, Ciudad Universitaria de Cantoblanco, 28049 Madrid, Spain; maria.martin@uam.es; 3 Instituto de Investigación en Ciencias de la Alimentación (CIAL-UAM-CSIC), Ciudad Universitaria de Cantoblanco, 28049 Madrid, Spain

Cardiometabolic diseases are one of the leading causes of morbidity and mortality worldwide, and the beneficial effect of diets rich in fruits and vegetables is widely recognized. Oxidative stress and inflammation are acknowledged as fundamental mechanisms implicated in the development and progression of these diseases, and the health benefits of plant-based diets against them could be related to the antioxidant and anti-inflammatory properties of the wide array of bioactive compounds they contain. The characterization of foods, food extracts, and specific phytochemicals, and the identification of their mechanism of action, is key to validating the relationship between diet and cardiometabolic health scientifically. This Special Issue addresses interdisciplinary research focused on foods with antioxidant properties, their potential benefits to counteract obesity, hypertension, diabetes and other related cardiometabolic diseases and the molecular mechanism implicated in their effects. 

Thirteen contributions have been selected for publication in this Special Issue, including research articles on human, animal and in vitro studies and reviews. Some of them have pointed out the relevance of lifestyle strategies, including dietary regimens enriched in phytochemicals with health-related properties, as first-line treatment of various cardiometabolic diseases. In the review of Nunes et al. [1], the authors focus on blueberries as a source of phytochemicals to prevent (pre)diabetes progression. They compile data ranging from in vitro assays to animal models and human studies, aiming to disclose the potential mechanisms implicated in the health-promoting properties of blueberries. The authors also draw attention to the need for additional efforts regarding studies with standardized interventions in humans that will hopefully bring more robust evidence and concrete guidance for blueberries’ effective use in prediabetes. In this line of work, Basu et al. [2] provide evidence of the effects of strawberry consumption in a randomized, controlled crossover study conducted in adults with features of metabolic syndrome. The authors report significant increases in serum antioxidant capacity and superoxide dismutase activity, together with decreases in lipid peroxidation and inflammatory biomarkers. These data also confirm that consuming strawberries for 4 weeks significantly improved antioxidant status, endothelial function, and inflammation in adults with cardiometabolic risks. Another example of the benefits of antioxidants to ameliorate the burden of cardiometabolic disease in humans is the research of Duc et al. [3]. The study analyzes a large Korean population of over 60,000 individuals and explores the relationship between metabolic syndrome, heavy metal levels, intake of antioxidant vitamins, and curry consumption. They found that vitamin B1, B2, B3, C, and A intakes were lower in subjects with metabolic syndrome, while they had higher levels of heavy metals. Additionally, they evidenced that the risk of metabolic syndrome was significantly lower for high curry consumers. Therefore, these results show the potential health benefits resulting from vitamins and curry intake to protect the population against cardiometabolic diseases. One of the manifestations of metabolic syndrome is non-alcoholic fatty liver disease, being that lifestyle is a core aspect of its development, particularly dietary patterns. The study by Martinez-Urbistondo et al. [4] explores a cohort of liver-disease-free patients in a clinical setting, the association between the FIB-4 index score, a non-invasive liver fibrosis scale, with lifestyle, in terms of antioxidant habits and quality of life. This study sets a proof of concept of the linkage between lifestyle and undetected oxidative liver fibrosis, supporting the relevance of antioxidant dietary patterns as an approach to prevent non-communicable disorders in the population. Together with oxidative stress, unresolved inflammation plays a critical role in cardiometabolic disease development. This is the focus of the review of Gila-Díaz et al. [5], which analyzes the role of specialized pro-resolving mediators (SPMs) derived from long-chain polyunsaturated fatty acids in resolving inflammation and generating tissue repair. Most of the studies have centered on the impact of SPMs in adult cardiometabolic health, and in this review, the authors address their role in neonates. Their study highlights the importance of nutrition in the early stages of life to prevent cardiometabolic disease development and the relevance of gaining knowledge on aspects such as SPM biosynthesis and receptor-mediated actions to develop novel pro-resolving therapies in neonates.

The second group of contributions in the Special Issue is dedicated to specific food-derived bioactive compounds. Over the past two decades, numerous experimental and epidemiological studies have shown that the consumption of flavonoid-rich foods reduces the risk of developing cardiovascular diseases. Among them, certain flavonoids, such as (−)-epicatechin and taxifolin, have demonstrated relevant antihypertensive properties and the capacity to improve endothelial function. The review of Bernatova and Liskova [6] focuses on the current knowledge of the above-mentioned phytochemicals, underlining the molecular mechanisms implicated and determined in preclinical studies. In addition to the attention on hypertension, the authors remark on the potential benefit of these flavonoids as antiviral substances, adding current knowledge regarding their possible synergic benefits in the treatment of hypertensive subjects with SARS-CoV-2 infection. In addition to their capacity to improve cardiovascular function, flavonoids also have health-promoting properties against metabolic alterations. This issue is the subject of the contribution of Prasatthong et al. [7], who demonstrate that galangin, a natural flavonoid, given to rats with metabolic syndrome induced by diet, alleviates cardiac alterations, oxidative damage, and inflammation. In another animal model of obesity, the Zucker rat, Dayar et al. [8] address the potential benefits of *Lonicera caerulea* L. (Loni), rich in anthocyanins as a promising source of beneficial polyphenols with therapeutical potential in cardiometabolic diseases. They demonstrate that Loni decreased LDL and total cholesterol levels, associated with increases in NOS activity and SOD expression and reduction in NADPH oxidase and NF-kappa B. Therefore, the authors propose that the improved lipid profile by the intake of Loni is related to the antioxidant effect of its phytochemicals. Another phenolic compound with cardiovascular effects is genistein, an isoflavone derived from soya beans, a food widely used in Asia, which possesses antihypertensive actions as demonstrated by Poasakate et al. [9] in a rat model of hypertension and cardiac dysfunction. In their work, they reveal that genistein given as a supplement at 80 mg/kg weight/day for 5 weeks exhibited cardioprotective effects, mediated by the suppression of oxidative stress through inhibition of the renin-angiotensin system. The Mediterranean diet is a dietary pattern with well-known health benefits due to its abundance of bioactive compounds from plant foods, being one of the hallmarks of the use of olive oil. The study of González-Hedström et al. [10] evaluates in aged rats the effects of a nutraceutical based on the addition of olive leaf extract to a mixture of algae and extra virgin olive oils. They demonstrate that this new nutraceutical may be a good strategy to treat and/or prevent the metabolic, cardiovascular, and muscle alterations associated with aging through the reduction in inflammatory and oxidative stress markers.

This Special Issue also incorporates a study about the cardiovascular effects of supplementation with the symbiotic formulation Prodefen® on a rat model of hypertension. The contribution of Méndez-Albiñana et al. [11] reveals the beneficial effects of this food, reducing high blood pressure in a genetic model of hypertensive rats through an increased neuronal NO release and an enhanced antioxidant effect, mediated by an increased Nrf2/Cu-Zn SOD activation.

Finally, the issue contains two articles about the revalorization of industrial by-products as an approach that could contribute to human health and environmental protection. They focus on cocoa shells, studying their phytochemicals in vitro and ex vivo in the context of cardiometabolic diseases. The study of Rebollo-Hernánz et al. [12] describes, in HepG2 human hepatocytes, the effects of a cocoa shell extract in the activation of FGF21 signaling, the inhibition of oxidative stress and mitochondrial dysfunction, and in the modulation of lipid and glucose metabolism. They demonstrate that phytochemicals from cocoa shells, especially protocatechuic acid, can trigger this signaling pathway attenuating inflammation, oxidative stress, lipid accumulation, and glucose intolerance in the above-mentioned cell culture. In addition, in rat arteries, this cocoa shell extract exhibits vasodilatory effects with higher potency compared to classical endothelium-dependent vasodilators. In the study by Rodriguez-Rodríguez et al. [13], the authors demonstrate that these actions are related to its antioxidant phytochemicals, particularly protocatechuic acid, which protects NO from degradation by superoxide anion. In addition, cocoa shell extract was able to improve relaxation in age-induced endothelial dysfunction. The valorization of this by-product entails innovative approaches to generate novel food ingredients and zero waste.

We are pleased to present this Special Issue that provides current knowledge in the research area of antioxidant foods and cardiometabolic health. We want to thank all the authors who have contributed by sharing their research and the implicated reviewers for their work to improve the published articles. We hope that the readers will find this Special Issue encouraging and a source of inspiring new ideas for future research in this expanding field.
